# Drowsiness Detection Based on Intelligent Systems with Nonlinear Features for Optimal Placement of Encephalogram Electrodes on the Cerebral Area

**DOI:** 10.3390/s21041255

**Published:** 2021-02-10

**Authors:** Seunghyeok Hong, Hyun Jae Baek

**Affiliations:** 1 Division of Data Science, The University of Suwon, Hwaseong-si 18323, Korea; shongdr@gmail.com; 2 Department of Medical and Mechatronics Engineering, Soonchunhyang University, Asan 31538, Korea

**Keywords:** EEG, biosignal, measurement, sleepiness, fatigue, DDS, driving, machine learning, random forest, SVM

## Abstract

Drowsiness while driving can lead to accidents that are related to the loss of perception during emergencies that harm the health. Among physiological signals, brain waves have been used as informative signals for the analyses of behavioral observations, steering information, and other biosignals during drowsiness. We inspected the machine learning methods for drowsiness detection based on brain signals with varying quantities of information. The results demonstrated that machine learning could be utilized to compensate for a lack of information and to account for individual differences. Cerebral area selection approaches to decide optimal measurement locations could be utilized to minimize the discomfort of participants. Although other statistics could provide additional information in further study, the optimized machine learning method could prevent the dangers of drowsiness while driving by considering a transitional state with nonlinear features. Because brain signals can be altered not only by mental fatigue but also by health status, the optimization analysis of the system hardware and software will be able to increase the power-efficiency and accessibility in acquiring brain waves for health enhancements in daily life.

## 1. Introduction

Drowsiness is defined as the transitional state of falling asleep. The brain in the drowsy state has yielded to the slowness or cessation of body movement, as indicated by the etymology of drowsiness, such as “drūsian” and “dreosan” in Old English, which mean “sink” and “to become slow”. The state of the brain yields negative outcomes without an adequate self-awareness of global indicators such that driving in a drowsy state can lead to unintended damage, injury, and death, especially in view of the fact that automotive improvements in recent times have not considered safe in an adequate manner [1]. Unfortunately, many people continue to drive despite their self-awareness of sleep deprivation and its risks [2]. Additionally, people easily underestimate the severity and risk caused by drowsiness [3]. However, sleep-deprived people are not able to assess situations to prevent the occurrence of accidents.

Brain waves have been used to investigate and understand drowsiness based on variations of consciousness [4,5,6]. The electrophysiological signals measured on the scalp are known as electroencephalograms (EEG). The accumulated knowledge from prior research efforts based on EEG and sleep stage analyses offer basic insights for the EEG features required to assess different states of drowsiness. Brain waves have been analyzed based on the delta (<4 Hz), theta (4–7.99 Hz), alpha (8–12.99 Hz), beta (13–30 Hz), and gamma (>30 Hz) spectra bands [7,8,9]. A number of additional information, including time-domain and nonlinear features, have been used to analyze different stages of sleep [10,11,12,13]. These EEG features have been used for feature extraction in almost all studies of drowsiness detection. However, the protocol used for physiological data lengths to analyze each research approach has not been standardized [14]. The degree of drowsiness changes with respect to time in daily life, based on variations in physical and mental conditions.

Additionally, due to the advances in mobile EEG systems [15] (e.g., lightweight hardware, wireless transmission, and software), we can evaluate the optimal electrode location for specific tasks. This evaluation of a limited sensor installment is required because additional electrodes can cause more discomfort for wearers. According to a comparative review of seven mobile EEG devices, few studies considered user discomfort [16]. Comfort of use and visual appearance with limited electrode number should be considered when designing a practical system.

Unlike other EEG studies that focused solely on increasing detection ability, we focused on the locations of the electrodes, considering the specific characteristics of the stages of drowsiness and the information quantity. We inspected the outcome of varying the data length for each EEG channel for a broader outlook on transitional drowsiness to find the effective cerebral area. The variety of data lengths that determine the quantity of the prior information for training can affect the feature characteristics and performance of the signals used for the detection of participant drowsiness. Therefore, drowsiness was detected using EEG with a combination of time, frequency, complexity, and entropy features affected by data length variations. We derived nonlinear features from each separate spectral range of EEG produced by band-pass filtering. After the feature extraction, we used supervised machine learning methods, random decision forest (RF), and support vector machine (SVM). The RF is a robust ensemble method that does not suffer from overtraining [13,17]. The terminology ‘random’ was influenced by the idea of searching over a random subset when splitting a node or random subspace selections [18]. The SVM has been used for drowsiness classification based extension of solution surfaces from linear to non-linear and use of optimal hyperplanes with support vectors [19,20].

The remaining sections are organized as follows. Section 2 describes the materials and methods for the EEG measurements during the virtual driving simulation. The feature extraction and RF machine learning processes were presented to classify states of drowsiness. Section 3 consists of descriptions of the findings from experiments for the estimation of drowsiness. Section 4 includes discussions on the results for EEG-based drowsiness detection system (DDS). Finally, Section 5 summarizes the conclusions of the preceding sections.

## 2. Materials and Methods

### 2.1. EEG Acquisition during Driving Tasks in Simulations

**Figure 1:**
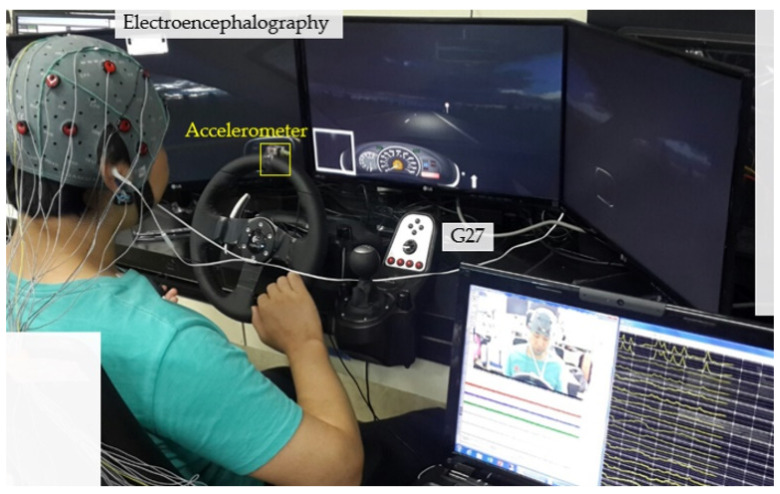
Driving simulation with electroencephalogram (EEG) measurements and video recording.

Virtual driving at a fixed speed (e.g., 95 km/h) on a straight highway was set as the fundamental task. The monotony of driving in a separate and quiet space for 55–70 min with no other cars or pedestrians was used to induce drowsiness. The driving simulation software (Carnetsoft B.V., Groningen, The Netherlands) displayed a three-dimensional (3-D) in-vehicle view of a driver on three 24-inch monitors (Figure 1).

To monitor reactions to the vehicle lane-keeping task, we asked participants to control a G27 steering wheel (Logitech, Lausanne, Swiss Confederation). The virtual world was created with a width limit that allowed a drowsy vehicle operator to realize the proper direction for keeping the lane. Participants only used their visual senses without any environmental sound to avoid unexpected auditory stimulation effects. Forced lateral moves of the car (e.g., 5 km/h to the left or right) over random intervals (5–19 s) occurred in scenarios for behavioral analyses. The video for the offline annotation of the state of the upper body and steering wheel was recorded by a video camera, LifeCam HD-5000 (Microsoft, Redmond, WA, USA) in front of the driver.

**Figure 2:**
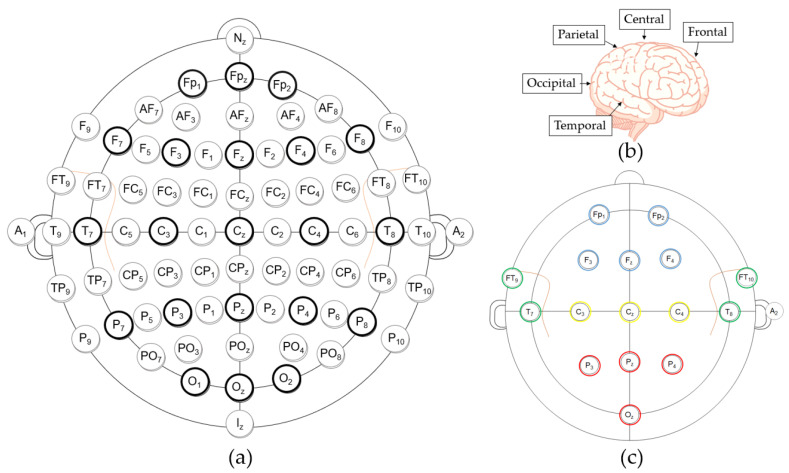
(**a**) The 10-10 electrode placement system (Bold: 10–20 system). (**b**) The cerebral lobes and central area. (**c**) The placement configuration of the 16 electrodes.

Standard 16-channel EEGs were acquired by a g.USBamp device (g.tec Medical Engineering, Schiedlberg, Austria) with a 24-bit quantization level at a sampling rate of 512 Hz during the simulation. The locations of the Ag/AgCl electrodes were selected on the basis of the international 10-10 electrode placement system [21], as illustrated in Figure 2a. The abbreviations in Figure 2a denote the following: the frontal (F), temporal (T), parietal (P), and occipital (O) cerebral lobes, and the central (C) area around the vertex in Figure 2b, where “z” symbolizes “zero” on the midline between the nasion (N) and the inion (I). For grouping, electrodes on the posterior area (i.e., Pz, P7, P8, and Oz) were symbolized as PO. The subscripted numbers of single electrodes indicate the left (odd) or right (even) hemisphere of the brain and the relative distance from the zero line.

To achieve balanced measurements of all cerebral areas, the electrodes were positioned at Fp1, Fp2, Fz, F3, F4, Cz, C3, C4, T7, T8, FT9, FT10, Pz, P7, P8, and Oz, as shown in Figure 2c. The ground electrode was placed at Fp_z_, and a clip-type reference electrode was fixed at A2. A bandpass filter (0.1–50 Hz) was used to decrease noise and a 60-Hz notch filter was used to diminish the power line interference in the hardware.

For the EEG and video synchronization, participants were asked to turn the steering wheel in the clockwise and counterclockwise directions at the beginning and end of the experiment. The steering motion was measured by a 3-axis accelerometer fixed on the uppermost position of the steering wheel (Figure 1). The acceleration data were recorded by a video camera and an MP150 data acquisition system (BIOPAC Systems Inc., Goleta, CA, USA) at a sampling rate of 1 kHz. For accelerometer and g.USBamp recordings, the BIOPAC system was synchronized by an ATMEGA128 microcontroller unit (ATMEL Co., San Jose, CA, USA) based on 5-V pulses with 1-s duration that were sent to the inputs of both systems.

### 2.2. Participants and Experimental Conditions

A total of 16 (12 men and 4 women) healthy right-handed participants (25–32 years old) participated in the EEG measurements for the driving simulation experiments. The research was approved by the Institutional Review Board (IRB) of the Seoul National University Hospital (IRB No. C-1509-074-704). All participants provided informed consent before they engaged in the study. The voluntary agreement included the control of the intake of chemicals, such as not drinking caffeinated drinks (for 5 h) and alcohol (for 24 h) before the experiment to avoid factors that affect drowsiness. Considering the possibility of drowsiness suppression by hunger, experiments were conducted 40–70 min after meals. The physical activity of the participants was not considered, and no extraneous physical activities (e.g., labor or exercise) were reported on the day before the experiment. To learn how to handle the steering wheel and avoid health issues during the simulation, the participants were allowed to experience preliminary drives for 5 min. During the pretest, no one felt 3-D simulation sickness.

### 2.3. Feature Extraction with Signal Processing

**Figure 3:**
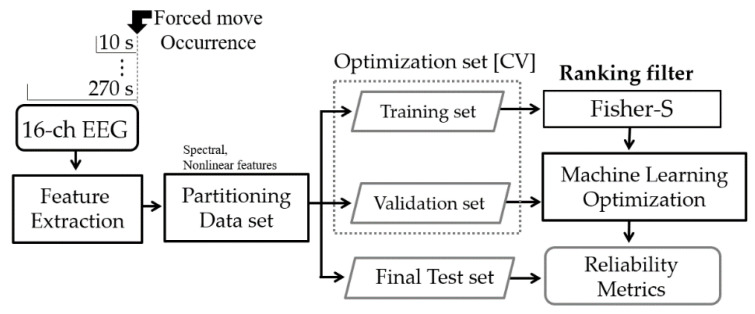
Machine learning procedure using electroencephalograms (EEG).

The acquired signals were analyzed using MATLAB R2020a (MathWorks, Natick, MA, USA). All EEG signals were resampled at 512 Hz and normalized. The accelerometer data recorded with the EEG and the videos were synchronized based on the steering wheel movements for cues. We recorded data for 60 s in advance of the start time of the experiments because the first 60 s of data must be excluded to eliminate the cue movements for synchronization and transitional waveforms from all filtered signals. Then, the EEG features were calculated for all data length options before every forced move event, as shown in Figure 3.

Spectral features were derived using the periodogram, which is a nonparametric estimate of the power spectral density (PSD) with a Hanning window in the frequency domain. The power, frequency, gravity frequency [10], and frequency variability [20] of the five basic spectral features were calculated for all EEG frequency bands. In addition, the ratios between the pairs of band powers (i.e., a total of six combinations from the bands) were derived. A total of 26 features in the frequency domain could be used.

We conducted feature augmentation on the basis of knowledge about physiological functions in EEG. Nonlinear features were computed for each separate signal in terms of delta, theta, alpha, and beta spectral ranges after digital band-pass filtering (i.e., Butterworth). Four nonlinear features for four spectral bands yielded a total of 16 nonlinear features. The procedure allowed comparison of the outcomes yielded by spectral features and by nonlinear features.

The generalized Hurst exponent (H) and Higuchi fractal dimension (HFD) were computed. The exponent H, named after Hurst, is a statistical measure that represents the signal tendencies with time. The Hurst exponent was generalized using the first-order moment of the increment distribution, as detailed in the algorithm described in a previous report [22]. The H value ranged between 0 and 1 and contained information about the signal on the basic criterion. H = 0.5 indicated that the signal exhibited random walk patterns (i.e., a Brownian time series). H values > 0.5 represented more persistent processes related to low fractal dimensions (FDs). Conversely, the FD was derived based on Higuchi’s algorithm [23]. The FD is a representative measure that quantifies the ratio of the change in a fractured signal pattern to a change in the scale.

The number of time series *N* can be described as

(1) $$X_{k}^{m}:x\left(m\right),x\left(m+k\right),x\left(m+2k\right),\ldots ,x\left(m+\left[\frac{N-m}{k}\right]\cdot k\right)$$

for the initial time *m* = 1, 2, …, *k*, where the interval time is *k* = 2, …, *k*_max_. In this case, [] represents the Gauss’ notation, and both *k* and *m* are integers.

The curve length $L_{m}\left(k\right)$ is estimated using Equation (2).

(2) $$L_{m}\left(k\right)=\left\{\left(\overset{\left[\frac{\left(N-m\right)}{k}\right]}{\underset{i=1}{\sum }}\left|x\left(m+ik\right)-x\left(m+\left(i-1\right)k\right)\right|\right)\frac{N-1}{\left[\frac{N-m}{k}\right]\cdot k}\right\}/k$$

The length of the curve for the time interval *k* can be estimated using the arithmetic mean of $L_{m}\left(k\right)$ for all *m* values defined as 〈$L_{m}\left(k\right)$〉 or L(k). Therefore, the HFD is identified as the angular coefficient of the linear regression of the graph of *ln*(*L*(*k*)) versus *ln*(1/*k*) using the linear least-squares fitting procedure [24] applied to the pairs of (*k*, *L*(*k*)) by varying *k* (*k*_max_ = 8 in the present study).

Furthermore, we applied the viewpoints of entropy in the frequency and time domains. The spectral entropy (SpEn) [25], the entropy of the PSD, was derived in the normalized form as

(3) $$SpEn=\left(-\overset{f_{U}}{\underset{i=f_{L}}{\sum }}P_{i}\log P_{i}\right)/N_{f}$$

where *f_L_* and *f_U_* are the lower and upper frequencies of a band, respectively, *P_i_* is the relative power (the ratio of each power to the total power) over the frequency *i*, and *N_f_* is the number of frequencies within the band. The permutation entropy (PmEn) was derived by symbolizing the elements of a time series as the defined permutation patterns. For the time series ${\left\{x_{t}\right\}}_{t=1,\ldots ,T}$, we defined *n* different encoding numbers that yield *n*! possible permutations *π* of order *n* [13]. An encoded permutation list for time series group was sequenced in increasing order of the values of the matched *n*-time series (*n* = 4 and *lag* = 1). The raw data for the PmEn were resampled at the sampling rate of 64 Hz in the present study. For a counted number denoted as #, the relative frequency *p*(*π*) for each permutation *π* is defined as

(4) $$p\left(\pi \right)=\frac{\#\left\{t|t\leq T-n,\left(x_{t+1,\ldots ,}x_{t+n}\right)has\;type\;\pi \right\}}{T-n+1}$$

The entropy associated with the distribution of the relative frequency (*PmEn*) is then defined as

(5) $$PmEn=-\sum^{\;}p\left(\pi \right)\log p\left(\pi \right)$$

where the sum includes *n*! permutations *π* of order *n*. These features sequenced by a ranking filter (i.e., generalized Fisher score [26]) were utilized as input data for the following machine learning method to detect the state of drowsiness.

### 2.4. Classification of Drowsiness

The ground truth used for statistical analysis with respect to drowsiness was labeled by researchers experienced in sleep analysis and behavioral observations on the 10 s videos around each forced move [13]. To assign true sleepiness labels, behavioral measures of percentage eyelid closure, head pose, and yawning rate have been widely utilized. The drowsiness descriptions were divided into ‘awake’ and ‘drowsy’ classes through omission of the median of the five levels of the European Transport Safety Council report in 2001 [27]. The levels of ‘not drowsy’ and ‘slightly drowsy’ were grouped into the ‘awake’ class, and the ‘very drowsy’ and ‘extremely drowsy’ were grouped into the ‘drowsy’ class. The actual labels were confirmed through an agreement among all three researchers. We utilized an RF that compounded the results of different decision trees that developed their branches and internal nodes by using roots (prior information) and leaves (detections). The generalization ability of the RF could be explained by randomizing the training steps to fuse the characteristics of each tree by bootstrap aggregating (bagging) [18]. The bagging algorithm randomly selects a subset of detectors for growing each tree. We selected 128 trees to form the forest, which we considered to be an appropriate number of trees based on the biological data [28]. We adapted SVM using the radial basis function kernel, which has shown robust results in studies of drowsiness and sleep stage classifications [25,29]. The hyperparameters to optimize the SVM model were selected by the random search method [30] implemented in MATLAB 2020a.

**Figure 4:**
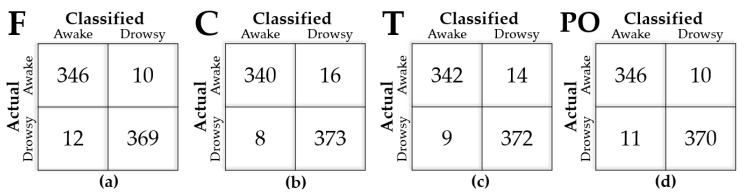
Confusion matrix yielded by random decision forest (RF) with a 180-s EEG on all features of cerebral areas of (**a**) frontal, (**b**) central, (**c**) temporal, (**d**) posterior.

A reliability analysis was performed using a random division of the optimization set (two-thirds) and the final test set (one-third). To avoid biased results, the cross-validation (CV) process was performed for the optimization set, which was repeatedly separated into training sets (e.g., 4/5 of the optimization set) and validation sets (e.g., 1/5 of the optimization set). The minimum mean-squared-error of classification of the fivefold CV defined the condition for an optimal number of ranked features. The performance was represented by the accuracy of classified labels by the intelligent system relative to the ground truth labels. The task achievements of drowsy detections were yielded from unseen 737 cases of forced move events by confusion matrix results as shown by an example in Figure 4. Accuracy was calculated as the number of identical cases of actual and classified labels divided by the number of total cases for each class in percentage [%].

### 2.5. Comfort Rating Scale of Measurements on Cerebral Areas

Participant discomfort due to electrode placement on a cerebral area was evaluated on a 0-to-10 numerical rating scale based on a visual analog scale (VAS). The two comfort descriptors ‘perceived change’ and ‘anxiety’ were assessed using a conventional comfort rating scale (CRS) [31]. The VAS is the most common scale for quantification of discomfort in medical studies [32,33]. The participants were asked to report discomfort using the VAS (where 10 stood for maximum discomforts) after familiarizing themselves with the criteria of Wong-Baker FACES pain scales, which matched a numerical scale to six facial expressions [34].

## 3. Results

The average number of forced movement events in driving simulations was approximately 390 for all participants. The average number of annotated states of drowsiness was approximately 47, with a standard deviation (SD) of 22 for all participants. The number of awake and drowsy states was balanced for each participant.

**Figure 5:**
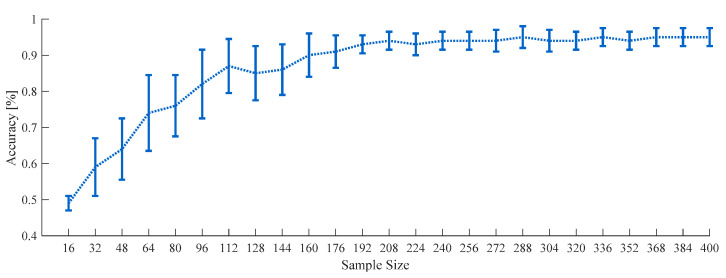
Learning curve of RF depending on total sample size using all features from 180-s data on 16-ch full-scalp EEG with standard deviation (SD) bar.

The appropriate sample size for machine learning classifications could be determined by fitting a classifier learning curve produced using empirical data as described in a study about inverse power-law models [35]. The previous study showed that learning classifier learning curves generally follow the inverse power law. This alternative inspection with an increase of sample size has been used like a statistical power consideration in studies using hypothesis tests [36]. A total of 184 cases among all participants (14 cases of each participant) successfully fitted learning curves for both RF and SVM models, as in Figure 5.

### 3.1. Single Electrode Placement for EEG-Based DDS

Generally, we realized that the reliability yielded from RF and SVM approached a plateau at data lengths of 180 s for all electrode cases. Table 1 and Table 2 list the reliability outcomes with two different amounts of information in the RF and SVM modeling. The increase of accuracy by including nonlinear features with spectral features was symbolized as Δaccuracy on the basis of spectral feature results.

If we focused on the results of the spectral features, Fp1 and Fp2 exhibited the highest values for 30-s EEG and 180-s EEG using RF results and for 30-s EEG using SVM. The variations in the data length could offer a variety of insights based on considerations of the changeable character of the state of drowsiness. The electrodes on the F area exhibited high performance in both data quantity cases using spectral features.

We observed that 16 nonlinear features derived from four EEG bands achieved drowsiness classification accuracies similar to the results achieved using 26 spectral features. Feature augmentation based on the knowledge of physiological functions can provide information on forest growth or identification of optimal hyperplanes. Except Fp1, all results using nonlinear features of 30-s data were superior to those using spectral features.

The accuracy of all areas was improved by the combination of nonlinear features with spectral features for both machine learning methods. Using 30-s EEG, the improved results showed an average difference of 2.9% of RF and 4.17% of SVM, respectively. Increasing the data length from 30 to 180 s produced accuracy differences of 1.16% of RF and 0.97% of SVM.

When using all spectral and nonlinear features, accuracy for all participants showed a difference of 6.93% in RF and 5.54% in SVM when increasing data length from 30 to 180 s. In SVM results, this difference was about 59% smaller than that between the results of the 30- and 180-s spectral features only (9.08%). The additional information showed positive effects in terms of reliability. The nonlinear features offered supplementary insights to the machine learning for shorter data lengths. In addition, the sensitivity to data length (i.e., from 30-s to 180-s) can vary depending on the use of spectral, nonlinear, or all features, with accuracy differences of 9.09%, 6.57%, and 5.55, respectively.

**Figure 6:**
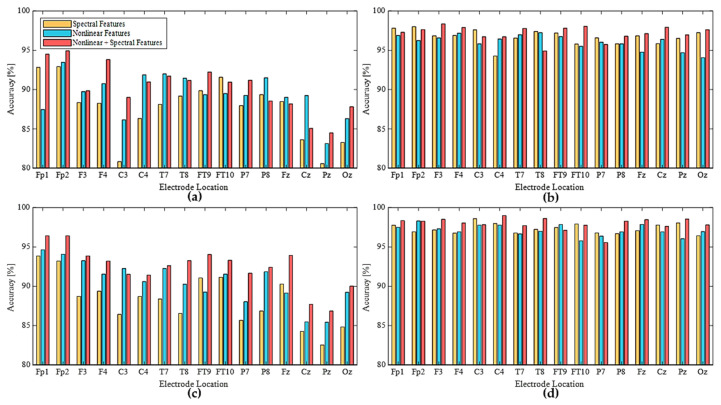
Classification accuracy using spectral and nonlinear features with intelligent systems: (**a**) RF [30-s], (**b**) RF [180-s], (**c**) SVM [30-s], (**d**) SVM [180-s].

Figure 6 shows the representative classification accuracies yielded by RF and SVM intelligent systems for drowsy states using spectral and nonlinear features of every EEG electrode. The overall tendencies of RF and SVM were similar to each other in terms of information quantity. In 30-s data on the frontal area, the combination (3rd bar) of nonlinear and spectral features achieved much higher performance than the others. For single-electrode location, every electrode on the central line symbolized as ‘z’ produced lower accuracy than the other electrodes. This result could be related to lateralization of brain processing of basic sensory information in left and right hemispheres. The neural signals from the lateral sides of the cerebral lobes could be divided into left and right sides based on the affected body parts [37]. Therefore, an electrode on the interhemispheric fissure of the central longitudinal line might contain a smaller amount of drowsiness information than others. This is because evidence of drowsiness is based on a sensorimotor function decrease that causes slower reactions.

For classification performance using all features, we could select an optimal location of a single electrode for EEG utilized for DDS. For the participants in the present study, an electrode on the frontal lobe including Fp1, Fp2 (best for 30-s data), or F3 (best for 180-s data) was optimal. When the EEG system had sufficient availability to operate multiple channels for DDS, we grouped the features of multiple channels for each cerebral area (Figure 2c) to create an input dataset.

### 3.2. Cerebral Area for EEG-Based DDS

Figure 4 presents an example confusion matrix derived by RF with 180-s data of grouped channels to cover the cerebral areas. SVM using each dataset from F, C, T, and PO areas yielded the accuracy of 97.01%, 96.74%, 96.88%, and 97.15%, respectively. All cerebral areas showed an accuracy greater than 96%. If we consider a strict criterion for the highest performance, F and PO areas could be selected. In following analyses about grouped electrodes, we focused on the SVM results, as they were superior to RF results in general.

By taking advantage of multiple channels, more informative pattern analyses could enhance the performance of the detected data. Table 3 lists the reliability with three feature sets (i.e., spectral, nonlinear, and combination) based on the EEG data of each cerebral area. For example, upon the feature combination of the electrodes on a specific area with 30-s spectral features, the accuracy increased from 91.07% (i.e., average value for single-electrode results on F) to 95.95% by grouping electrodes on the F lobe, as in Table 3. The electrodes on the F lobe showed outstanding performances up to a 60-s data length, like the 30-s result of a single electrode.

The grouping effect was outstanding for nonlinear features, with higher accuracy in 30-s, 60-s, and 90-s data compared to the results of spectral features. The number of nonlinear features was much lower (i.e., 40) than the number of spectral features when considering the four sub-bands of EEG.

In most cases, the combination of spectral and nonlinear features achieved the highest accuracy compared to the result by only spectral or nonlinear features in cerebral areas. With composite features from longer data, the reliability of the electrodes on the T lobe and the PO areas increased and was greater than that of the F area. The nonlinear and combined features on the T lobe classified drowsy states with an accuracy of 97.6% (90-s) and 99.13% (180-s) that are similar to the best result using all electrodes. The performance of electrodes on P and O areas was 99.25% (210-s), while that of all electrodes was 99.4% (270-s). However, in terms of data length variation, the F lobe showed consistent performance, with accuracies of 98.23% (150-s), 98.5% (180-s), and 99.07% (210-s).

The spectral information from the spread of the brain wave to the hemispheres outperformed that from the C area, like the results of a single electrode. However, the nonlinear features enhanced the information at longer data lengths and compensated for the lack of spectral information in the C area.

### 3.3. Comfort Rating of Electrode Placement

**Figure 7:**
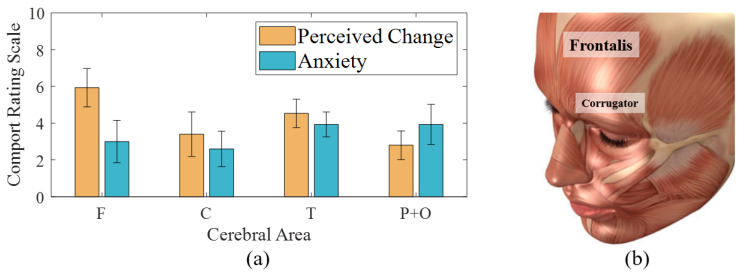
(**a**) Comfort rating results about electrode placements on cerebral area, (**b**) Frontalis and corrugator supercilia of human face muscles [Stocktrek Images. Anatomy of human face muscles. Digital Image. Alamy. Alamy Stock Photo, 1 May 2013. Web. 13 January 2021.

Figure 7a shows the mean and SD of rated ‘perceived change’ and ‘anxiety’ of electrode use. The discomfort related to the perceived change varied depending on placement and individual preference, with an average distribution ranging from 2.8 to 5.93 with SD of 1.54 to 2.4. The highest mean perceived change was observed on the F lobe because participants were not used to object placement on the forehead. Namely, constraints of using frontalis muscle that covers the forehead can produce perceived change when opening the eyes. This is because the frontalis muscle (Figure 7b) of the F area has connections with the corrugator supercilii (i.e., muscle close to the eye) and attachments to the skin of the eyebrows (without bony attachments). Moreover, the corrugator is connected to the orbicularis oculi close to the eyelids. In other words, the electrodes on the frontalis muscle could cause awkwardness in eye and eyebrows movements for gaze controls used to drive. The rating of electrodes on the T lobe was 4.5 mainly because of high tension yielded by the cap fixation method using a chin strap (Figure 1). The innate characteristics of conventional EEG based on elasticity of cap fabrics affect the overall pressure of electrodes. In addition, higher pressure on the lateral electrodes could be produced by the material of the chin strap, which is less stretchable than are EEG cap materials. Therefore, the perceived change on the T lobe could be lessened by an alternative form of fixation [15]. For example, the use of in-ear EEG is possible considering the signal similarity to that of the temporal lobe and its feasibility in drowsiness studies [13,38].

Anxiety from electrode detachments showed a more even distribution than perceived change, with an average range from 2.6 to 3.93 and SD of 1.33 to 2.31. In addition, the values lower than the median value of CRS indicated non-severe anxiety during the one-hour measurement. However, anxiety about the contact stability had an inverse relationship with stiffness. The lowest anxiety was produced by the electrode on C because the central area of the human skull can support electrodes without attachment materials. The lower anxiety on F could be related to its lower steepness than that of T lobe and posterior areas despite individual differences in head shape. We considered the trade-off relationship between discomfort and classification performance to determine optimal locations of electrodes.

## 4. Discussion

Many previous studies of drowsiness using low-cost EEG systems [39] have focused on the spectral features from a fixed information system. However, our study demonstrated the utility of nonlinear features and of various data length observations for analysis with limited electrodes to minimize user discomfort.

**Figure 8:**
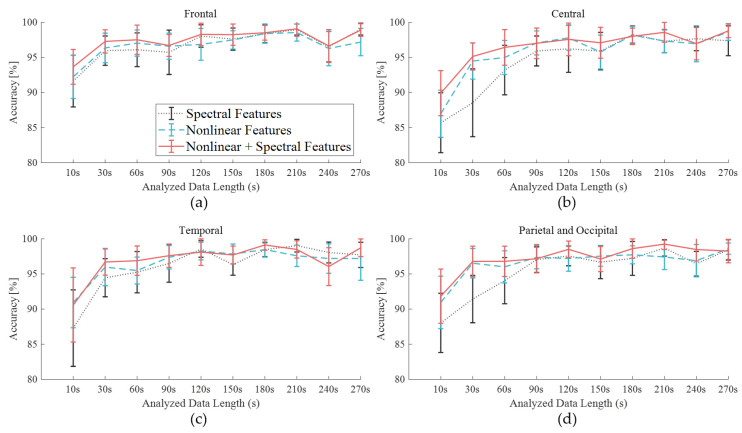
Accuracy derived by SVM with the data length variation using the spectral features only and the composite features with nonlinear features of cerebral area: (**a**) frontal, (**b**) central, (**c**) temporal, (**d**) posterior.

Figure 8 shows the SD of accuracy for all participants in the three datasets of spectral features (dotted line), nonlinear features (dashed line), and an extended dataset with nonlinear features (solid line). The C area had the highest SD, which meant it had the most inconsistent results across participants, although it showed the lowest CRS (Figure 5) when focusing on discomfort.

Due to the inclusion of the nonlinear features, both an increase in accuracy and greater consistency of the results yielded by the frontal and posterior areas were observed. The greater information quantity produced a lower SD for the sets containing nonlinear features compared to those containing only spectral features. However, longer data lengths did not always produce higher accuracy values after arrival at the plateau, such as in the case of the F area with a 180-s data length. The importance of information quantity could vary by cerebral area. The shorter data string for the F lobe and the longer data string for the PO lobes yielded informative features. The shorter data string for the F lobe and the longer data string for the PO lobes yielded informative features. These results were consistent with previous vigilance studies that considered cerebral areas. In a vigilance estimation study, researchers reported that the posterior (12-ch) EEG contained critical information in comparison with temporal (6-ch) EEG and forehead electrooculogram [40]. Among 19 EEG channels in an auditory vigilance task, the 10 top-ranked features comprised six features on the posterior area, two features on F, and two features on T lobes [41]. The T lobe features were highly ranked in the present study as well, with a result of 99.13% (180-s). If a new form factor could reduce the discomfort caused by structural characteristics of head shape, T and posterior areas could be selected for optimal placement of electrodes.

Some channel locations in the specific cerebral area were researched in previous EEG-based DDS studies using machine learning methods, as shown in Table 4. Commonly based on feature extractions by tunable Q-factor wavelet transform for the 3 channels (C3-O1, C4-A1, and O2-A1) of 16 participants in MIT-BIH polysomnographic database, a hybrid model of long short-term memory (LSTM) with pretrained models (e.g., AlexNet and VGG16) [42] and an extreme learning machine (ELM) [43] yielded accuracies of 94.3% and 91.8%, respectively. Using a five-channel EEG (C3, P4, P7, O1, and O2) with a support vector machine (SVM), an accuracy of 76.4% was demonstrated [44]. These studies with limited electrodes commonly observed the C and O areas. The result of our RF for the P and O lobes was similar to that of a deep learning method, although we should note the dataset difference.

On the other hand, full scalp EEG (approximately 30 EEGs) were previously tried to maximize the classification performance for detection of drowsiness. The performance of the Bayesian neural network with an independent component analysis (ICA) produced an accuracy of 88.2% for 43 participants [45]. Adaptive boosting (AdaBoost) of the decision tree classifier (maximum depth = 9) yielded an accuracy of 97.5% for 28 participants [46]. A back-propagation neural network showed an accuracy of 98.3% for 12 participants [12]. Our result with a 16-channel EEG achieved a similar level of results as the ~30-channel EEG due to the ensemble of nonlinear features and the varying information quantity.

For the daily measurement for EEG-based DDS with the optimal electrode placements, the electrodes on O and T lobe could be achieved with ear-set type hardware. Considering the robustness of shorter data for monitoring drowsiness, the inclusion of electrodes on F and P lobes could be possible with a hair band type system without electrodes on the C area, unlike a general-purpose EEG headset, which covers the full scalp [15].

## 5. Conclusions

The present study used a 16-channel EEG with an RF to demonstrate the detection of drowsiness with varied spectral and nonlinear information quantities. The main contribution of this study was the demonstration that optimal single and multiple electrodes on specific cerebral areas could be selected to minimize the discomfort of the DDS users. Few EEG studies using grouped multiple electrodes on cerebral areas have been reported regarding efficiency in DDS. Many studies for wearable EEG systems have focused on the enhancement of learning methods after selecting a location of a single electrode [47,48]. To find a better form factor for each brain structure and for broader insight, nonlinear features derived from sub-bands of EEG and variations of the data length were considered in the present study. The reliability values for every signal electrode increased as the data lengths increased, and these values were stable when the data length was approximately 180 s. The present results might be limited under laboratory conditions, which had no electrical artifacts or interruptions that occur in real environments. The performance might be limited in our dataset because the number of participants was not statistically sufficient.

Despite these limitations, the results are meaningful because the research target was to overcome the low accessibility of EEG, which has been a fundamental obstacle to the accumulation of big data. For real-life measurements out of the lab, it would be difficult to sustain the quality of EEG that affects the classification performance for transitional states [1]. In this regard, the utilization of nonlinear features like PmEn is worth consideration when it is difficult to acquire stable data. In terms of the feature curation, we could utilize the variation in detection performance of the cerebral area with data length. In the participant data, the optimal performance of the DDS systems had longer data lengths on the P and O lobes. While considering individual differences, the higher performance by each channel or area selection could enhance the accessibility of the EEG-based DDS to avoid false alarms. With advances in the mobile system to enhance user comfort [49], EEG could be an accessible tool not only for safety but also for health enhancements. It is expected that optimization analysis of the system will contribute to the advances in monitoring brain functions for the digital healthcare [50] or brain–computer interface for patients [51] by increasing the power-efficiency and accessibility for acquiring brain waves.

## Figures and Tables

**Table 1:** RF classification accuracy [%] using spectral and nonlinear features derived from 30- and 180-s data from each electroencephalographic (EEG) electrode.

Ch.	30-s EEG				180-s EEG			
	S ^1^	N ^2^	S + N	Δaccuracy ^3^	S	N	S + N	Δaccuracy
Fp1	92.84	87.48	94.51	1.67	97.83	96.89	97.29	0.54
Fp2	92.94	93.47	94.94	2.00	98.03	96.26	97.64	0.39
F3	88.35	89.74	89.86	1.51	96.86	96.59	98.38	1.52
F4	88.26	90.74	93.82	5.56	96.90	97.19	97.93	1.03
C3	80.84	86.16	89.01	8.17	97.60	95.82	96.73	0.87
C4	86.33	91.88	90.99	4.66	94.28	96.45	96.73	2.45
T7	88.12	91.99	91.72	3.60	96.58	97.00	97.81	1.23
T8	89.17	91.45	91.19	2.02	97.40	97.24	94.92	2.48
FT9	89.85	89.36	92.23	2.38	97.20	96.77	97.83	0.63
FT10	91.57	89.51	90.97	0.60	95.79	95.51	98.07	2.28
P7	87.97	89.26	91.21	3.24	96.59	96.06	95.74	0.85
P8	89.36	91.51	88.55	0.81	95.81	95.82	96.80	0.99
Fz	88.48	89.01	88.18	0.30	96.86	94.76	97.13	0.27
Cz	83.60	89.24	85.06	1.46	95.84	96.42	97.96	2.12
Pz	80.59	83.12	84.51	3.92	96.52	94.69	96.98	0.46
Oz	83.26	86.32	87.82	4.56	97.24	94.06	97.63	0.39
Mean	87.60	89.39	90.29	2.90	96.71	96.10	97.22	1.16

^1^ S: Spectral features; ^2^ N: Nonlinear features; ^3^ Δaccuracy: Accuracy difference by including N from results of S.

**Table 2:** Support vector machine (SVM) classification accuracy [%] using spectral and nonlinear features derived from 30- and 180-s data from each electroencephalographic (EEG) electrode.

Ch.	30-s EEG				180-s EEG			
	S ^1^	N ^2^	S + N	Δaccuracy ^3^	S	N	S + N	Δaccuracy
Fp1	93.84	94.62	96.41	2.57	97.74	97.50	98.33	0.59
Fp2	93.21	94.07	96.41	3.20	96.92	98.30	98.26	1.34
F3	88.70	93.24	93.86	5.16	97.15	97.31	98.51	1.36
F4	89.36	91.53	93.19	3.83	96.74	96.93	98.04	1.30
C3	86.43	92.25	91.51	5.08	98.58	97.77	97.82	0.76
C4	88.68	90.58	91.43	2.75	97.96	97.78	98.98	1.02
T7	88.37	92.24	92.61	4.24	96.76	96.66	97.71	0.95
T8	86.57	90.25	93.24	6.67	97.23	96.98	98.61	1.38
FT9	91.04	89.25	94.03	2.99	97.46	97.84	97.11	0.35
FT10	91.14	91.53	93.29	2.15	97.90	95.78	97.75	0.15
P7	85.67	88.02	91.65	5.98	96.79	96.37	95.55	1.24
P8	86.86	91.83	92.40	5.54	96.67	96.91	98.28	1.61
Fz	90.26	89.14	93.93	3.67	97.06	97.86	98.46	1.40
Cz	84.28	85.48	87.69	3.41	97.79	96.91	97.61	0.18
Pz	82.53	85.43	86.86	4.33	98.04	96.03	98.54	0.50
Oz	84.83	89.22	90.01	5.18	96.40	96.94	97.80	1.40
Mean	88.24	90.54	92.41	4.17	97.32	97.12	97.96	0.97

^1^ S: Spectral features; ^2^ N: Nonlinear features; ^3^ Δaccuracy: Accuracy difference by including N from results of S.

**Table 3:** Accuracy [%] using SVM with spectral and nonlinear features derived from 30- to 180-s EEG on cerebral areas.

Feature	Area	30 s	60 s	90 s	120 s	150 s	180 s
S ^1^	F ^3^	95.95	96.08	95.72	98.03	97.60	98.36
	C	88.54	93.19	95.91	96.22	95.89	98.24
	T	94.46	95.25	96.49	98.56	96.34	98.46
	PO	91.43	94.04	97.04	97.57	96.68	97.22
	All	95.87	97.43	97.91	98.30	97.19	98.52
N ^2^	F	96.34	97.03	96.59	96.85	97.48	98.38
	C	94.48	94.98	97.05	97.78	95.76	98.17
	T	95.98	95.49	97.38	98.26	97.84	98.47
	PO	96.53	96.01	97.37	97.21	97.54	97.73
	All	97.33	98.00	97.83	97.74	98.39	98.28
S + N	F	97.27	97.50	96.69	98.27	98.23	98.50
	C	95.13	96.42	97.00	97.56	97.08	97.99
	T	96.70	96.89	97.60	98.11	97.70	99.13
	PO	96.78	96.80	97.17	98.51	97.12	98.61
	All	97.52	97.78	97.78	98.82	97.37	99.20

^1^ S: Spectral, ^2^ N: Nonlinear, ^3^ F: Frontal; C: Central; T: Temporal; P: Parietal; O: Occipital areas.

**Table 4:** Arithmetic mean of statistical measures of performance in EEG-based detections of drowsiness.

Author	Representative Classifier	Channel # (Area)	Accuracy [%]	Participants # (age)
Chai et al.	Bayesian Neural Network	32	88.2	43 (18–55 years)
Hu et al.	Adaptive Boosting	30	97.5	28 (19–24 years)
Min et al.	Back propagation Neural Network	30	98.3	12 (19–24 years)
Awais et al.	SVM	5 (C3, P4, P7, O1, O2)	76.4	22 (18–35 years)
Umit et al.	Pretrained AlexNet, VGG16 + LSTM	3 (C3-O1, C4, O2)	94.3	16 (average: 43 years)
Bajaj et al.	ELM	3 (C3-O1, C4, O2)	91.8	
Present study	SVM	4 (T7, T8, FT9, FT10) 4 (Oz, Pz, P3, P4) 16	99.13 (180-s) 99.25 (210-s) 99.4 (270-s)	16 (25–32 years)

## Data Availability

Data sharing not applicable. The data are not publicly available due to participants’ privacy.
